# Genome sequence of the yeast *Candida sake* UCD2293, isolated from soil in Ireland

**DOI:** 10.1128/mra.01410-25

**Published:** 2026-03-16

**Authors:** Matthieu Osborne, Sean J. Aherne, Sean A. Bergin, Emma Flannery, Conor Hession, Tim Jordan, Heather Kershaw, Paula Mercade Lopez, Anna O'Connor, Ugne Ramanauskaite, Tadhg Ó Cróinín, Kenneth H. Wolfe, Geraldine Butler, Kevin P. Byrne

**Affiliations:** 1 School of Medicine, Conway Institute, University College Dublin 37438, Dublin, Ireland; 2 School of Biomolecular and Biomedical Science, Conway Institute, University College Dublin 637815, Belfield, Ireland; University of Maryland School of Medicine, Baltimore, Maryland, USA

**Keywords:** yeasts, genome analysis

## Abstract

*Candida sake* is a member of the Debaryomycetaceae family of budding yeasts. We present the genome sequence of *C. sake* strain UCD2293, isolated from soil at Poolbeg, Co. Dublin, in Ireland. This genome is 14.26 Mb and was assembled into 8 chromosome-sized contigs plus a mitochondrial genome contig.

## ANNOUNCEMENT

*Candida sake* (1) is a yeast species that is a member of the Debaryomycetaceae family of budding yeasts (2). It was first isolated from sake-moto (3) and has been used as a postharvest biocontrol agent to prevent mold growth in pome fruit (4). Often misidentified, and with poor growth at temperatures of 30°C or above, it is unlikely that it plays much of a role as a mammalian pathogen (5). Three other genome assemblies are available for this species, including one from the type strain NRRL Y-1622 (2).

**Fig 1:**
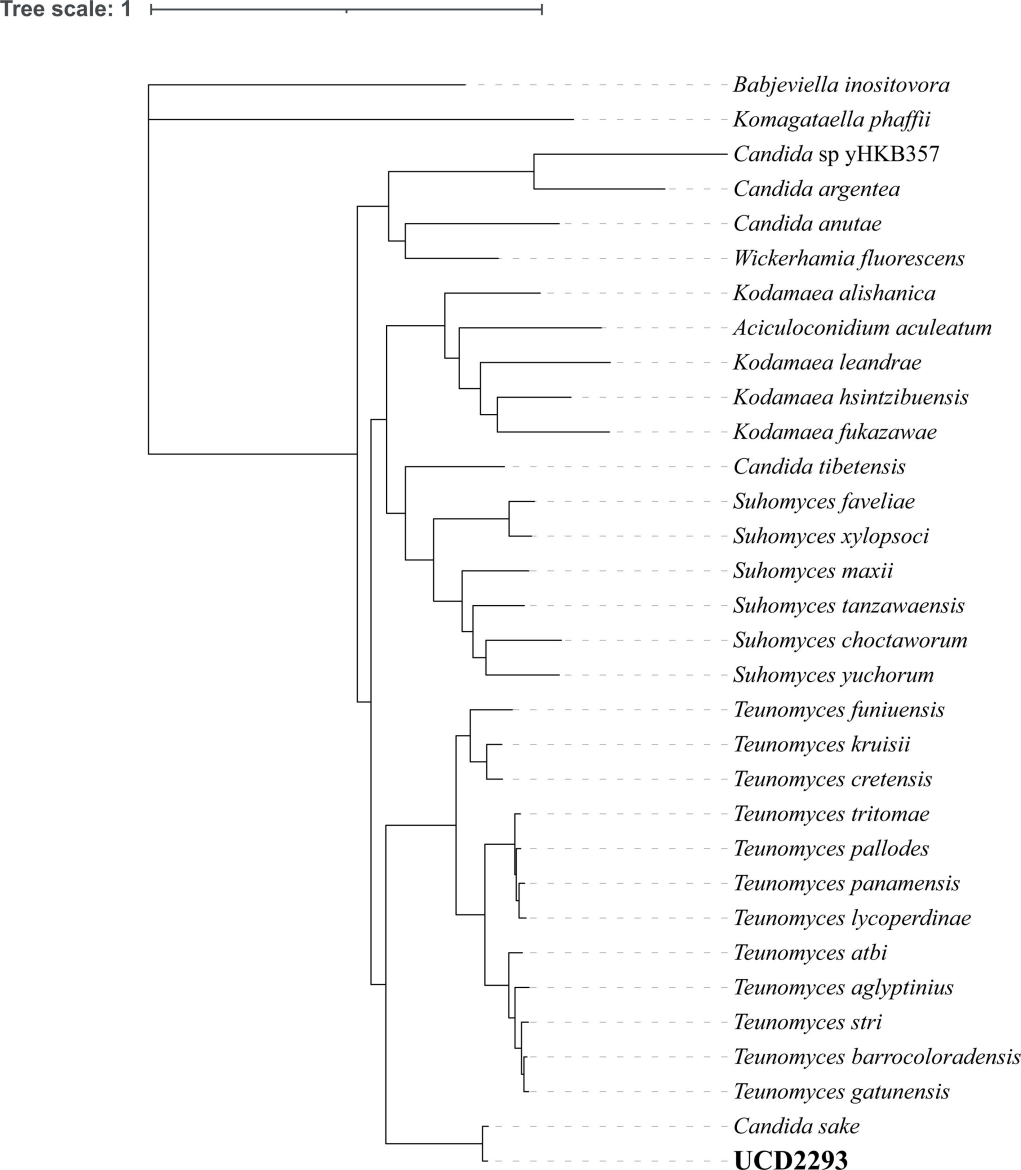
Phylogenomic tree. Phylogenomic analysis was performed on the basis of single-copy orthologs using short-read assemblies from UCD2293, 29 closely related species identified by BLAST searches of the UCD2293 rDNA locus, and two outgroup species *Babjeviella inositovora* and *Komagataella phaffii*. AUGUSTUS (version 3.5.0) was used to annotate the genome assemblies of all strains, with “--species=saccharomyces_cerevisiae_S288C” for *Komagataella phaffii* and “--species=candida_albicans” for the remaining strains (9). Subsequent proteins were concatenated and filtered to exclude those shorter than 100 amino acids and highly degenerate proteins with more than 10 stop codons annotated. Single-copy orthologs (SCOs) were identified by finding orthogroups using proteinOrtho (version 6.0.34) with basic parameters (10) and then filtering to keep only the 820 groups that comprise exactly 1 protein from all 32 strains. Each SCO group was aligned using MAFFT (version 7.526) with settings “--genafpair” and “--maxiterate 1000” (11), and alignments were trimmed using trimAl (version 1.2rev59) with the “-gappyout” parameter (12). Initial trees were found for each SCO group using RAxML (version 8.2.12) with the “PROTGAMMAAUTO” setting (13). SCO groups whose tree did not place the two outgroup species *B. inositovora* and *K. phaffii* outside of the other strains were discarded. The remaining 758 SCO group alignments were concatenated, and the final tree was generated using RAxML with 100 bootstrap iterations and parameters “-m PROTGAMMAALG -p 12345 -x 12345.” All branches in the final tree have 100% bootstrap support.

*C. sake* UCD2293 was isolated as part of an undergraduate research module (6) from soil collected from the soil bed approximately 5 meters from the start of the beachline at Poolbeg, Co. Dublin, Ireland (GPS coordinates 53.3370761–6.2101068). Alfalfa, turnip weed, and wild teasel were present. The soil material was passaged twice at room temperature in 9 mL liquid yeast extract-peptone-dextrose (YPD) containing chloramphenicol (30 μg/mL) and ampicillin (100 μg/mL) and cultured on YPD agar plates. The species was identified by PCR and Sanger sequencing of the ribosomal DNA internal transcribed spacer (ITS) and D1/D2 regions (accession numbers PX481660 and PX481661), using primers ITS1 (TCCGTAGGTGAACCTGCGG) and ITS4 (TCCTCCGCTTATTGATATGC) (7), and for the D1/D2 region using primers NL1 (GCATATCAATAAGCGGAGGAA) and NL4 (GGTCCGTGTTTCAAGACGG) (8). Sequence identity was 97.72% (387/396 bp) in the ITS region and 99.29% (560/564 bp) in the D1/D2 region, to the type strain of *C. sake* (accession numbers NR_151807 and KY106745). Phylogenomic analysis supports the identification (Fig. 1).

DNA was isolated by phenol/chloroform extraction from liquid YPD cultures grown at room temperature. Short-read library preparation and sequencing were performed by Novogene (UK) Company Ltd., using an Illumina NovaSeq X Plus platform, yielding 21.8 million read pairs (2 × 150 bp). Genomic DNA was randomly sheared into short fragments. The obtained fragments were end-repaired, A-tailed, and further ligated with the Illumina adapter. The fragments with adapters were PCR-amplified, size-selected, and purified. The library was checked with Qubit and real-time PCR for quantification and bioanalyzer for size distribution detection. Quantified libraries were pooled and sequenced. The library kit used was Novogene’s internal Novokit. Adapters and low-quality reads were removed (Skewer v0.2.2) (14). For long-read sequencing, after DNA extraction (MasterPure Yeast DNA Purification Kit MPY80010) and library preparation (Native Barcoding Kit SQK-NBD114-24) without shearing, we selected DNA >3 kb with Long Fragment Buffer, before Oxford Nanopore sequencing (MinION MK1B, flowcell FLO-MIN114) and Dorado “Super-ccurate” (SUP) basecalling (MinKNOW v24.06.16; default demultiplexing; barcode trimming; reads ≥ 500 bp kept; no quality cut-off). NanoFilt (v2.3.0) (15) retained 48,475 reads with quality ≥ 10 and length ≥ 1,000 bp (reads N50 = 24,406 bp), which were then assembled using Canu (v2.2) (16), followed by 5 rounds of error correction using NextPolish (v.1.4.1) with the Illumina reads (17). Default parameters were used, except where otherwise noted.

The assembly consists of 8 nuclear contigs (total 14.23 Mb; N50 = 2,036,733 bp) and the mitochondrial genome (27,246 bp circular unit; accession number CDRNKH010000009). All 8 nuclear contigs appear to be complete chromosomes because every end of every contig is either a probable telomere repeat sequence (TGATGATCTTTTACTACGGCGG)_n_ (18) or rDNA arrays (detected by BLASTN) (Table 1). All rDNA arrays are oriented with the 5S genes transcribed toward the nearby contig end.

**TABLE 1:** Genome assembly characteristics of *Candida sake* UCD2293

Chromosome	Size (bp)	Left end	Right end
1	3,665,350	Telomere	Telomere
2	2,081,437	Telomere	rDNA (5S only) → telomere
3	2,036,733	Telomere	Telomere
4	2,001,408	Telomere	Telomere
5	1,493,952	rDNA	Telomere
6	1,374,842	Telomere	rDNA → telomere
7	1,013,201	rDNA	rDNA → telomere
8	567,137	Telomere	Telomere
Mitochondrial	27,246	n/a (circular)^*a*^	n/a (circular)^*a*^

^*a*^ n/a, not applicable to a circular chromosome.

The UCD2293 genome assembly has an average nucleotide identity (19) of 91.17% to the assembly (accession no. JAJMAA010000000) of its closest relative, the *C. sake* type strain NRRL Y-1622 (2) (Fig. 1). Using BUSCO v5.8.2 (20), genome completeness was estimated as 97.1%, compared to the Ascomycota lineage data set. The G+C content is 38.84%.

## Data Availability

This whole-genome shotgun project has been deposited as DDBJ/ENA/GenBank accession number CDRNKH010000000 (BioProject no. PRJEB97774). The version described in this paper is version 1. The reads were deposited at the ENA (accessions ERR15761576 and ERR15761577). The ITS sequence is PX481660. The D1/D2 sequence is PX481661.
